# A multicenter study: New cut‐off values of antitransglutaminase antibodies processed by chemiluminescence in children with suspected celiac disease

**DOI:** 10.1002/jpr3.12169

**Published:** 2025-02-03

**Authors:** Gonzalo Ortiz, Florencia Ursino, Fernando Battiston, Gabriela Messere, Veronica Busoni, Rosana Solis Neffa, Roman Bigliardi, Mabel Mora, Maria del Carmen Toca, Marina Orsi

**Affiliations:** ^1^ Pediatric Gastroenterology Unit, Hospital Nacional Alejandro Posadas Buenos Aires Argentina; ^2^ Pediatric Gastroenterology Unit, Hospital Italiano de Buenos Aires CABA Buenos Aires Argentina; ^3^ Pediatric Gastroenterology Unit, Hospital Materno Infantil de San Isidro San Isidro Buenos Aires Argentina

**Keywords:** antitissue transglutaminase antibody, celiac diagnosis, intestinal biopsy

## Abstract

**Objectives:**

(1) To assess the predictive value of anti‐tissue transglutaminase immunoglobulin A (IgA) antibodies (a‐tTG) by chemiluminescence immunoassay (CLIA) related to duodenal histology in children with suspected celiac disease (CD) and (2) to determine the cut‐off value of a‐tTG by CLIA that allows diagnosis of CD avoiding biopsy.

**Methods:**

Retrospective, descriptive, observational study in children between 1 and 16 years of age, studied for CD. Patients with IgA deficiency and those on a gluten‐free diet were excluded. Sensitivity (S), specificity (Sp), positive predictive value (PPV), and negative predictive value (NPV) were calculated for ≥10, 30, and 50 times the normal a‐tTG (normal value [NV]) compared with histology, evaluated by blinded pathologists.

**Results:**

The total number of patients included was 262. The a‐tTG IgA by CLIA with a cut‐off point of 20 chemiluminescent units (CU) had a S of 99.5%, Sp, 10.26%, PPV, 86.38%, NPV, 80%. When a cut‐off value of a‐tTG ≥ 10 times NV (200 CU) was used, the S, Sp, PPV, and NPV were 65.4%, 69.23%, 94.2%, and 25.96%, respectively. Likewise, with a value ≥30 times NV (600 CU) the correlation with histology was 99.03%, reaching a PPV of 100% with a cut‐off value ≥50 NV (1000 CU). Combining both methods a‐tTG IgA + EMA IgA, we obtained similar results to the a‐tTG IgA level for the proposed cut‐off values.

**Conclusions:**

We suggest that the use of 30 times the NV cutoff values would be the best predictor of which patients do not need a biopsy.

## Abstract (highlights)

**What is Known:**

- There is a correlation between immunoglobulin A (IgA) antitransglutaminase level and histological findings in celiac disease (CD).
- The European Society for Paediatric Gastroenterology Hepatology and Nutrition (ESPGHAN) 2020 diagnostic guidelines have established that in children who have anti‐tissue transglutaminase IgA antibodies (a‐tTG) more than 10 times above normal value and positive anti‐endomysial antibodies (EMAIgA) in a 2nd serum sample, regardless of symptoms, the duodenal biopsy can be avoided
- The cut‐off values used in the ESPGHAN (ESPGHAN 2020) diagnostic guidelines are based on enzyme‐linked immunosorbent assay (ELISA) methodology.
- Currently, many centers around the world use the chemiluminescence methodology due to its higher efficiency for sample processing, decreasing the diagnostic time.

**What is New:**

- It is necessary to apply different cut‐off values for anti‐tissue transglutaminase IgA antibodies (a‐tTG) by chemiluminescence methods because they differ from those proposed for ELISA methodology.
- We determine new cutoff values to avoid a small bowel biopsy for CD diagnosis.

## 1 INTRODUCTION

Celiac disease (CD) diagnosis relies mainly on anti‐tissue transglutaminase immunoglobulin A (IgA) antibodies (a‐tTG) levels to determine the need of duodenal biopsy.^1^, ^2^ a‐tTG antibodies have a recombinant human transglutaminase as a substrate. They have a high sensitivity (S) and specificity (Sp) and can be processed by enzyme‐linked immunosorbent assay (ELISA) or chemiluminescence immunoassay (CLIA) techniques. Instead, endomysial antibodies (EMA IgA) are detected by indirect immunofluorescence performed on monkey esophageal epithelium. As it is an expensive and operator‐dependent method, it is mainly used as a confirmatory test.^3^, ^4^ In recent years, many centers around the world have opted to use the CLIA technique due to its highly efficient sample processing and lower cost.

Duodenal biopsy is considered a “gold standard” for the diagnosis of CD. Pathology findings are villous atrophy, cryptic hyperplasia, and the presence of increased intraepithelial lymphocytes.^5^, ^6^ Several studies have shown a good correlation between a‐tTG titers and duodenal histological involvement.^7^, ^8^, ^9^ Based on those studies, in 2012, the European Society for Paediatric Gastroenterology, Hepatology and Nutrition (ESPGHAN) formulated new guidelines introducing the possibility of a “non‐biopsy” approach in a specific subgroup of pediatric patients. The criteria included symptomatic patients with compatible genetics, that is, the presence of at least one allele of the major histocompatibility complex (HLA) DQ2/DQ8, together with the determination of a‐tTG IgA antibody titers >10 times the upper limit of normal value (NV) and positive IgA endomysial antibodies in a second sample.^10^ Subsequently, based on more publications demonstrating the excellent correlation between serology and histopathological findings in 2020, a review of the ESPGHAN 2012 criteria was carried out providing new evidence on some aspects. The new guidelines postulated that symptoms and HLA need not to a determining factor in celiac diagnosis using nonbiopsy technique, in the cases in which a‐tTG IgA is >10 × NV, confirmed by the EMA in a second sample.^11^

A new methodology which was based on CLIA became available for the detection of CD‐specific autoantibodies. Although this methodology presented excellent sensitivity and specificity in preliminary studies,^12^, ^13^ it is necessary to validate its performance and analyze whether the same cut‐off values apply using CLIA, given the importance that this determination has in the new diagnostic ESPGHAN 2020 algorithm.^11^

The aim of our study was: (1) to assess the predictive value of atTG IgA antibodies by CLIA related to duodenal histology in children with suspected CD, and (2) to determine the cut‐off value of a‐tTG by CLIA that allows diagnosis of CD avoiding biopsy.

## 2 METHODS

A retrospective, descriptive, observational study was conducted in children aged 1–16 years, who were evaluated for suspected CD at three reference centers in Buenos Aires, Argentina between January 2018 and December 2021. All children with serological tests for CD performed by CLIA, and duodenal biopsies of the first and second portion obtained by upper digestive endoscopy were included. All patients were consuming gluten at the time of diagnosis.

Patients who had total IgA deficiency (IgA < 7 mg/mL) or who were on a gluten‐free diet at the time of diagnosis were excluded.

### 2.1 Duodenal biopsies

Two biopsies from the duodenal bulb and four from the second portion of the duodenum were obtained endoscopically. The biopsies were randomly assigned to pathologists, who were blinded to serological test results. In cases of discordance, biopsies were reviewed by a third pathologist.

Histological findings were reported according to the Marsh‐Oberhuber criteria.^4^, ^6^ A Marsh value of 2 and above was considered diagnostic of CD.^14^, ^15^

### 2.2 Laboratory tests

We analyzed serum from patients who underwent endoscopy for suspected CD for a‐tTG IgA using the CLIA method QUANTA Flash h‐tTG IgA, (BIO‐FLASH^®,^ Inova Diagnostics). A cut‐off value of ≤20 chemiluminescent units (CU) was considered normal. In addition, all patients had EMA determination using a commercially available indirect immunofluorescence kit using sections of monkey esophagus as substrate (NOVA Lite Monkey Esophagus IFA kit®, Inova Diagnostics), with a detection dilution of 1:10.

All samples were analyzed with the same kits using the same cut‐off values.

### 2.3 Statistical analysis

Qualitative variables were described with frequency and percentages. The quantitative variables were expressed with measures of central tendency and dispersion according to the distribution that it presented (normal: mean and standard deviation or non‐normal: median and interquartile range).

For the study of a diagnostic test, the sample size was calculated through the program Epidat 4.2 with the following specifications: for an expected sensitivity and specificity of 94%, and 97% respectively, with a confidence of 95% and a precision of 3%, a sample size of 242 patients was needed.

Sensitivity (S), specificity (Sp), positive predictive value (PPV), and negative predictive value (NPV) were calculated using the a‐tTG IgA and QUANTA Flash h‐tTG IgA CLIA results. Through receiver operating characteristic curves (ROC) and Youden's index, the best cut‐off points were established.

PPV: Probability that someone with a positive serologic test has histological CD.

Sensitivity, specificity, positive predictive values, and negative predictive values were calculated for ≥ 10, 30, and 50 times the normal a‐tTG.

#### 2.3.1 Ethics Statement

Informed consent and assent were obtained by legal guardians and patients as appropriate for age. The study was approved by the Ethics and Research Committee of Hospital Nacional A. Posadas (Date2022/No 607LMnPoSo/22) and validated by the ethics committees of each participating center.

## 3 RESULTS

**Figure 1:**
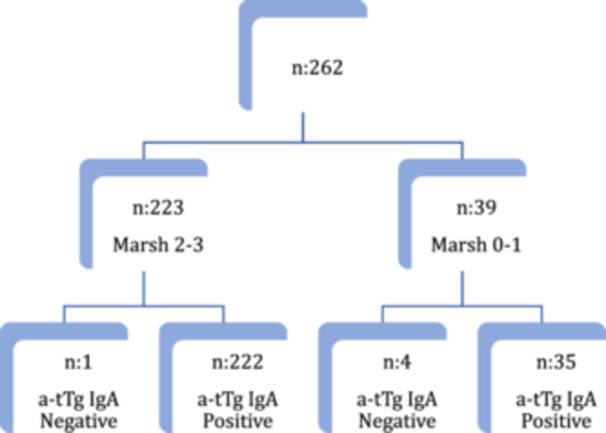
Graphic representation of patients distribution. a‐tTg, anti‐tissue transglutaminase; IgA, immunoglobulin type A.

A total of 262 patients were included in the study, 146 from the Alejandro Posadas National Hospital, 86 from the Italian Hospital of Buenos Aires, and 30 from the San Isidro Maternal and Children Hospital. The median age was 84 months (IQ‐1 range 48 m IQ‐3 132 m). Females *n*: 162 (66%). All patients had normal levels of total IgA. Thirty‐nine patients (14.47%) had a normal biopsy (Marsh 0/1). (Figure 1).

Two hundred twenty‐three patients (85.53%) had a biopsy compatible with CD (Marsh 2/3), of whom one (0.45%) had negative a‐tTG IgA but elevated DGP IgG ( ≥20 CU), 16 (7.17%) had titers less than three times the NV ( ≥ 20 to <60 CU), 57 (25.56%), had values between 3 and <10 times the NV (60 CU‐199 CU) and 149 (66.82%) had a‐tTG titer ≥10 times the NV ( ≥ 200 CU).

Of 39 patients with normal biopsy, a‐tTG IgA was negative in four ( ≤20CU) but three had another positive antibody, EMA IgA (+) and three DGP IgG ( ≥20CU). Thirty‐five patients had positive a‐tTg IgA, from which seven had a compatible genetic study, with normal endoscopic image and were considered as a potential CD. The remaining patients were assumed to be false positives.

The a‐tTG IgA by CLIA for the cut‐off point of 20 CU, showed sensitivity of 99.5%, specificity 10.26%, PPV 86%, NPV 80% for CD detection. However, due to the low specificity found, the best cut‐off point was using the Youden index, placing the a‐tTG IgA CLIA at 100 CU, with a S of 87.44%, Sp of 61.54%, PPV 92.86%, and NPV 46.15% (Table 1).

**Table 1:** Sensibility, specificity, and predictive values of atTG IgA QUANTA Flash with a cut‐off value of 20 CU and the best cut‐off point located at 100 CU.

Parameters	atTG IgA ≥ 20cu, %	LR 95%	atTG IgA ≥ 100cu, %	LR 95%
Sensitivity	99.55	(97.5, 99.92)	87.44	(82.45, 91.17)
Specificity	10.26	(4.061, 23.58)	61.54	(45.9, 75.11)
Positive predictive value	86.38	(81.65, 90.04)	92.86	(88.55, 95.62)
Negative predictive value	80	(37.55, 96.38)	46.15	(33.34, 59.5)

Abbreviations: a‐tTG IgA, anti‐tissue transglutaminase immunoglobulin type A; CU, chemiluminescent unit; LR, likelihood ratio.

**Figure 2:**
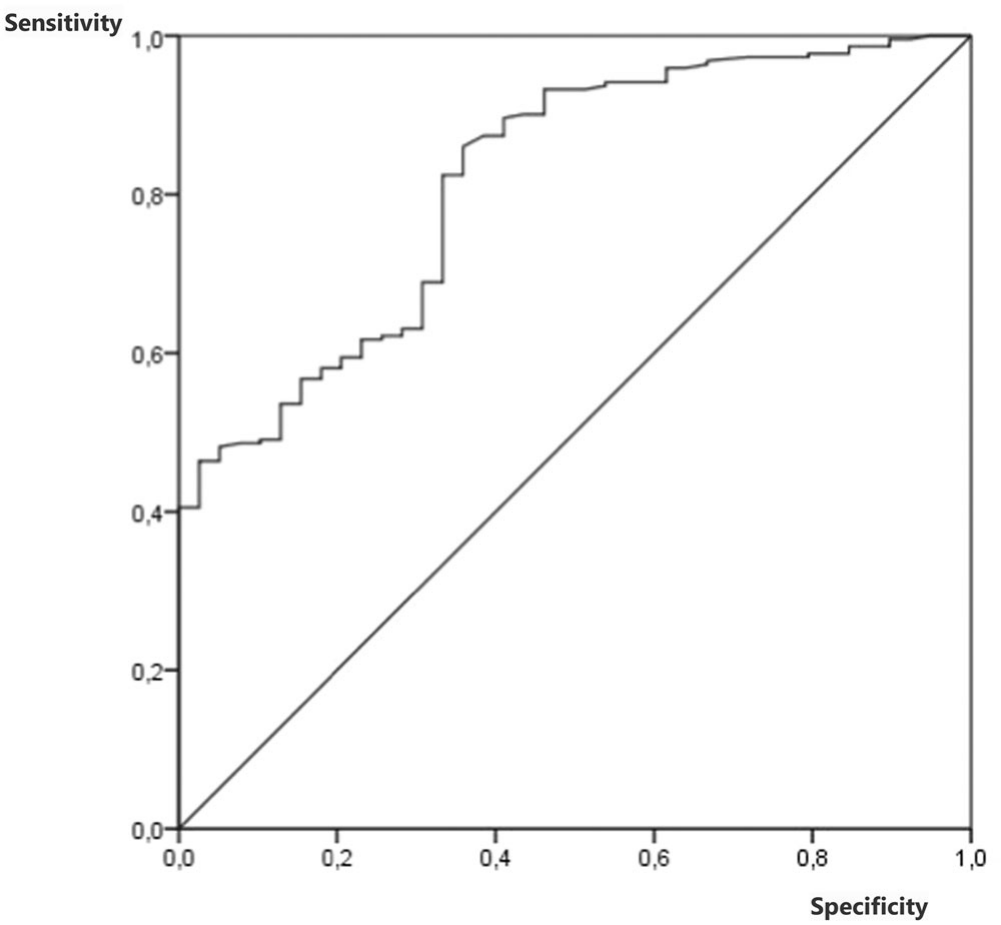
Curve ROC. ROC, receiver operating characteristic curves.

The area under the curve (AUC) was 0.745 with a 95% confidence interval (CI) 0.650–0.840. (Figure 2).

To evaluate the correlation between the results of the a‐tTG IgA assay and histopathological findings compatible with CD (Marsh 2–3) and, thus, to be able to identify the best cut‐off point to apply the new ESPGHAN 2020 diagnostic criteria, patients were divided into three subgroups according to the value obtained from a‐tTG IgA at ≥10, 30, and 50 times VN.^11^

Of all patients (262), 161 had a‐tTG IgA ≥ 10 NV (200 CU), whose S, Sp, PPV, and NPV were 68.16%, 69.23%, 94.2%, 27.55%. respectively. When a cut‐off value of ≥30 NV (600 CU) was used, correlation with histology (Marsh 2/3) was 99.03%, reaching a 100% PPV when the cut‐off value was ≥50 NV (1000 CU).

When results from a‐tTG IgA + EMA IgA were combined, the following results using cut‐off values of ≥10 NV (200 CU) were obtained: S 68.16%, Sp 71.70%, PPV 95.7%, NPV 26%. These results were similar to the a‐tTG IgA levels for the proposed cut‐off values, maintaining a PPV value of 100% at 50 times NV. (Table 2).

**Table 2:** Correlation between a‐tTg IgA cut‐off values and TGA‐IgA tests in combination with antiendomysium IgA and histopathological findings compatible with CD.

	Sensitivity %	**Specificity %**	PPV %	NPV %
Antitransglutaminase—cut off (*n* = 161)				
≥10 times normal value (200 cu)	68.16	69.23	94.2	27.55
≥30 times normal value (600 cu)	45.74	97.44	99.03	23.9
≥50 times normal value (1000 cu)	39.46	100	100	22.41
Antitransglutaminase + EMA IgA‐cut off (*n* = 161)				
≥10 times normal value (200 cu)	68.16	71.70	95.70	26
≥30 times normal value (600 cu)	45.74	97.44	99.03	23.9
≥50 times normal value (1000 cu)	39.46	100	100	22.41

Abbreviations: a‐tTG IgA, anti‐tissue transglutaminase immunoglobulin type A; CD, Crohns Disease; CU, chemiluminescent unit; EMA, anti‐endomysial; IgA, immunoglobulin type A; NPV, negative predictive value; PPV, positive predictive value; Se, Sensitivity; Sp, Specificity; TGA‐IgA, tissue transglutaminase IgA test.

## 4 DISCUSSION

The 2020 ESPGHAN guidelines for CD diagnosis indicate that children with 10 times above the cut‐off level of IgA a‐tTG with appropriate testing and positive EMA‐IgA on a second serum sample, would confirm the diagnosis of CD without the need of a biopsy. This new algorithm is based on the evidence that elevated a‐tTG values show a good correlation with histological lesions (Marsh grade 2 or 3).^8^, ^9^, ^13^, ^14^, ^16^

To establish a correct CD diagnosis mainly based on clinical signs and serology gives biochemical data a more relevant role in the diagnostic process. The disadvantages of this algorithm are the potential differences in the analytical performance due to variations in the processing methodology and the lack of standardization, enhancing the need of more prospective studies.^12^, ^17^

The ESPGHAN guidelines use ELISA as a serological method for the detection of a‐tTg IgA antibodies.^11^ In recent years, new serological methods for CD diagnosis have emerged, such as CLIA (e.g., QUANTA Flash h‐tTG IgA), and its implementation is a true revolution in the diagnosis of autoimmune diseases due to excellent S and Sp.

The CLIA a‐tTG IgA test (QUANTA Flash) presents a wide range of dynamic dispersion of the light beam, and results range from 0.1 to 4965.5 CU. The level of autoantibodies detected in the serum of patients with CD at the time of diagnosis is usually high.

The aim of a study by Previtali et al., in a population of children and adults, was to determine the predictive value of atTG by CLIA and its correlation with histology.^12^ Their findings showed a good performance of the antibody by CLIA where S and Sp at the cut‐off value of 20 CU were 98.2%, 98.4%, and a PPV of 97.9%. Regarding histological correlation, the PPV was 99% in children at 560 CU (28 times NV).^12^ In our study, the performance of ‐tTG by CLIA, with a cut‐off value of 20 CU showed S 99.5%, Sp 10.26%, PPV 86%, with a negative predictive value 80%.^18^, ^19^, ^20^, ^21^

The difference in the specificity of our study compared to that found by Previtali et al. may be due to the fact that they selected patients who had duodenal biopsies, regardless of the presumptive diagnosis, while our selection was based on those patients suspected of having CD. It may also be because their sample size was much larger than ours.

When we correlated histopathological findings (Marsh 2/3) with the a‐tTG titers, using different cut‐off values (10–30 and 50 NV), an adequate correlation was obtained, with positive predictive value for 30 and 50 times the NV of 99% and 100%, respectively. However, the value of 10 NV was not optimal for a‐tTG IgA CLIA method with a PPV of 94.2%, compared with histological lesions compatible with CD.

When the PPV was calculated at the different levels of a‐tTG IgA antibodies, we found that patients with >30 times the cutoff (600 CU) had a high probability of significant histological lesions (PPV 99%).

We found normal Marsh biopsy (0/1) in 39, and seven of them had positive serology (a‐tTg IgA) and HLA DQ2/DQ8 compatible with CD. Those patients are considered as potential CD according to ESPGHAN criteria. The rest of the patients were considered false positives.

In the study by Werkstetter et al.^6^ using cut‐of values of a‐tTGA‐IgA 10‐fold NV or more, a positive EMA, and the presence of any symptom, identified children with CD with a PPV of 99.75 (95% confidence interval [CI]). The authors also compared 8 a‐tTG platforms, including the CLIA method (QuantaFlash), and the combination with other serological tests such as EMA IgA. A diagnostic correlation of 100% PPV was observed with values of ≥10 times the NV in most of the tests analyzed, but in when chemiluminescence was used, a PPV greater than 99% was not achieved.

In our study, the combination of a‐tTG IgA + EMA IgA was analyzed, and no significant differences were observed in the diagnostic accuracy with the established cut‐off values for a‐tTG IgA alone.

## 5 CONCLUSIONS

We suggest modifying the cut‐off value of a‐TG according to the methodology used to process it because there are substantial differences between ELISA and chemiluminescence titers. Precision is even more important when CD diagnosis is performed without a confirmatory biopsy to avoid recommending a life with gluten‐free diet to patients who does not need it.

This allows intestinal biopsy to be omitted when the a‐tTG IgA values are ≥30 times the NV and a positive EMA IgA dosage.

It is important to be aware of this methodological difference when implementing biopsy‐free algorithms to avoid incorrect diagnosis of CD.

## CONFLICT OF INTEREST STATEMENT

The authors declare no conflicts of interest.
